# Advances in Systemic Therapy for Unresectable Hepatocellular Carcinoma: Commentary on The Impact of the STRIDE Regimen in HIMALAYA Trial

**DOI:** 10.32604/or.2026.069227

**Published:** 2026-02-24

**Authors:** Leenah Abdulgader, Abdullah Esmail

**Affiliations:** 1 Department of Surgery, University of Maryland Medical Center, Baltimore, MD, USA; 2 Section of GI Oncology, Houston Methodist Neal Cancer Center, Houston Methodist Hospital, Houston, TX, USA

**Keywords:** Hepatocellular carcinoma (HCC), single tremelimumab regular interval durvalumab (STRIDE) regimen, HIMALAYA trial, overall survival, systemic therapy

## Abstract

Unresectable hepatocellular carcinoma (HCC) remains a global challenge, with limited effective treatment options for advanced-stage disease. The HIMALAYA trial (phase III randomized study that evaluated the STRIDE regimen) introduced the Single Tremelimumab Regular Interval Durvalumab (STRIDE) regimen, an immunotherapy-based approach that achieved a median overall survival (OS) of 16.43 months compared to 13.77 months with sorafenib. While statistically significant, this ~2.7 months OS gain warrants scrutiny in light of STRIDE’s increased immune-related toxicity and cost. This commentary evaluates STRIDE’s impact within the broader landscape of first-line systemic therapy for unresectable HCC, alongside other regimens such as atezolizumab plus bevacizumab and nivolumab plus ipilimumab. We explore STRIDE’s mechanism of action, safety profile, modest progression-free survival (PFS) improvement, and implementation challenges, incorporating insights from 2023–2025 research. In addition, we discussed its limitations in non-viral HCC and Child-Pugh B patients, the role of emerging biomarkers, and the potential of radiation to enhance immunotherapy efficacy. As a dual immune checkpoint inhibitor (ICI) strategy, STRIDE offers an important advance that may not only extend survival but also open the door to future curative approaches. However, optimizing its use will require refined patient selection and further investigation of synergistic combination therapies.

## 1 Background and Importance

Primary liver cancer ranks as the sixth most common cancer and the third leading cause of cancer-related death globally [1]. According to updated 2024 GLOBOCAN estimates, its incidence has risen to approximately 900,000 new cases annually, with nearly 800,000 deaths [1]. Hepatocellular carcinoma (HCC) accounts for 75%–85% of primary liver malignancies, typically arising from hepatocytes in the setting of cirrhosis that is driven by chronic hepatitis B or C, aflatoxin exposure, heavy alcohol use, nonalcoholic fatty liver disease (NAFLD), obesity, type 2 diabetes, and smoking [2]. The Barcelona Clinic Liver Cancer (BCLC) staging system remains the most widely used framework for guiding HCC management. It incorporates tumor burden, liver function (via alpha-fetoprotein levels and albumin-bilirubin scores), performance status, and clinical symptoms to stratify treatment [2]. While early-stage HCC may be amenable to potentially curative interventions such as resection, ablation, or liver transplantation, but the majority of patients present with unresectable disease, where systemic therapies become the primary treatment modality [2].

The HIMALAYA trial introduced the Single Tremelimumab Regular Interval Durvalumab (STRIDE) regimen as a promising option to address this unmet need. By significantly extending overall survival compared to sorafenib, STRIDE represents a potential new standard in the first-line treatment of advanced HCC, and its clinical impact is further explored in this commentary.

## 2 Systemic Therapy for Unresectable HCC: Evolution over Time

Most patients with HCC are diagnosed at an advanced, unresectable stage, where systemic therapies aim to prolong survival and, in selected cases, downstage tumors for locoregional interventions like transarterial chemoembolization (TACE) or radiation therapy [3,4]. Understanding the evolution of these treatments provides essential context for the emergence of the STRIDE regimen as a first-line option [5].

Sorafenib is a multi-kinase inhibitor targeting vascular endothelial growth factor receptors (VEGFR), platelet-derived growth factor receptor β (PDGFR-β), and Raf kinases. It was approved in 2007 based on the SHARP trial [6]. This thereby inhibits angiogenesis and tumor proliferation. It delivered a median overall survival (OS) of 10.7 months, establishing a new therapeutic benchmark despite significant side effects such as hand-foot skin reaction and diarrhea [7].

In 2017, lenvatinib emerged from the REFLECT trial as a non-inferior alternative, offering a median OS of 13.3 months. Lenvatinib inhibits a broader array of targets, including fibroblast growth factor receptors (FGFR), RET, and KIT, though with adverse events such as hypertension and proteinuria [8,9]. The landscape shifted dramatically in 2020 with the advent of immunotherapy. The IMbrave150 trial combined atezolizumab (anti–PD-L1) with bevacizumab (anti–VEGF), to yield an OS of 19.2 months and a progression-free survival (PFS) of 6.8 months. This marked a pivotal move toward immune checkpoint inhibition [10].

In 2021, the COSMIC-312 trial introduced another combination—atezolizumab with cabozantinib, a multi-targeted TKI—achieving a median OS of 15.4 months. However, this regimen was associated with more complex toxicities, including hepatitis and hemorrhagic events [11].

Recent data, a 2024 real-world analysis [12], suggested that select combination regimens may now exceed 16 months of OS in well-defined patient populations, reflecting continuous refinement and personalization of therapy.

This trajectory—from monotherapy with TKIs to sophisticated immunotherapy combinations—culminates in the STRIDE regimen, which builds on these advancements to offer a new benchmark for overall survival in advanced HCC. Comparative details are summarized in Table 1.

**Table 1:** Summary of systemic therapies for unresectable

Therapy	Approval/Trial year	Median OS (Months)	Median PFS (Months)	Mechanism of action	Key adverse events
**Sorafenib [7]**	2007 (SHARP)	10.7	5.5	Multikinase inhibitor (VEGFR, PDGFR-β, Raf)	Hand-foot skin reaction, diarrhea
**Lenvatinib [8,9]**	2017 (REFLECT)	13.3	5.1	Multikinase inhibitor (VEGFR, FGFR, RET, KIT)	Hypertension, proteinuria
**Atezolizumab + Bevacizumab [10]**	2020, updated in 2022 (IMbrave150)	19.2	6.8	Anti-PD-L1 + anti-VEGF	Hypertension, fatigue, proteinuria
**Cabozantinib + Atezolizumab [11]**	2021 (COSMIC-312)	15.4	6.8	Anti-PD-L1 + multi-TKI (VEGF, MET, TAM)	Hepatitis, hemorrhagic events
**Nivolumab + Ipilimumab [13]**	CheckMate 040 (Cohort 4)	~22.8	~4.6	Anti–PD-1 + Anti–CTLA-4	Hepatitis, colitis, dermatitis, pneumonitis
**STRIDE (Tremelimumab + Durvalumab) [14]**	2022 (HIMALAYA)	16.43	5.4	Anti-CTLA-4 + anti-PD-L1	Immune-mediated events, hepatic issues

Note: Systemic therapies for unresectable HCC, evolution from TKIs to immunotherapy combinations. VEGFR, Vascular Endothelial Growth Factor Receptor; PDGFR-β, Platelet-Derived Growth Factor Receptor-β; Raf, Rapidly accelerated fibrosarcoma; FGFR, Fibroblast Growth Factor Receptor; RET, Rearranged during Transfection; Anti-PD-L1, Anti-Programmed Death-Ligand 1; anti-VEGF, Anti-Vascular Endothelial Growth Factor; multi-TKI, multi–tyrosine kinase inhibitor; TAM, Tumor-Associated Macrophages; Anti–CTLA-4, Anti–Cytotoxic T-Lymphocyte–Associated Protein 4.

## 3 STRIDE Regimen: A New Standard in Advanced HCC

The HIMALAYA trial_ a global, randomized, open-label, sponsor-blind phase III study, introduced the STRIDE regimen—a combination of tremelimumab (anti-CTLA-4) and durvalumab (anti-PD-L1)—as a promising new approach for patients with unresectable HCC [7], compared against sorafenib and durvalumab monotherapy in treatment-naïve patients. STRIDE demonstrated a statistically significant OS benefit of 16.43 months vs. 13.77 months with sorafenib, reinforcing its viability as a first-line therapy [7].

Approved by the FDA in October 2022, the regimen is administered as a single 300 mg dose of tremelimumab to initiate immune activation, followed by 1500 mg of durvalumab every four weeks, sustaining T-cell responses in the immunosuppressive tumor microenvironment typical of advanced HCC. Although the OS improvement over sorafenib (~2.7 months) is modest, the STRIDE regimen represents a critical mechanistic and clinical advance. However, it is associated with higher rates of immune-related adverse events and greater logistical complexity compared to tyrosine kinase inhibitors (TKIs). By shifting from targeted kinase inhibition to dual immune checkpoint blockade, STRIDE represents a paradigm shift in systemic HCC therapy. Its mechanism and clinical results—summarized in Table 1, support its role as a foundational option in the treatment landscape, offering durable responses through strategic immune priming [14].

Importantly, STRIDE is one of several effective front-line therapies, rather than a universally superior choice. For example, atezolizumab plus bevacizumab (A + B) achieved an updated OS of 19.2 months in the IMbrave150 trial, surpassing STRIDE’s survival benefit [15]. Similarly, nivolumab plus ipilimumab, another dual immune checkpoint inhibitor regimen, has shown high response rates and durable benefit in select patients. Thus, treatment selection should be personalized, considering efficacy, safety, patient characteristics, and underlying liver function.

## 4 Clinical Efficacy of the STRIDE Regimen

The HIMALAYA trial established STRIDE as a first-line therapy for unresectable HCC, demonstrating a median OS of 16.43 months (95% confidence interval (CI), 14.16–19.58) compared to 13.77 months (95% CI, 12.25–16.13) with sorafenib in treatment-naïve patients [7]. This nearly 3-month OS gain translated into higher survival rates at multiple time points: 48.7% (95% CI, 43.6–53.5) vs. 41.5% (95% CI, 36.5–46.4) at 18 months, 40.5% (95% CI, 35.6–45.3) vs. 32.6% (95% CI, 27.9–37.4) at 24 months, and 30.7% (95% CI, 25.8–35.7) vs. 20.2% (95% CI, 15.8–25.1) at 36 months for STRIDE and sorafenib, respectively [7].

In contrast, progression-free survival (PFS) showed no significant improvement, with STRIDE at 5.4 months (95% CI, 3.8–5.6) and sorafenib at 5.6 months (95% CI, 5.1–5.8). This lack of PFS benefit underscores the need to weigh OS gains against treatment-related toxicities, duration, and patient quality of life.

These findings reaffirm OS as the principal endpoint in unresectable HCC, where extending survival may enable downstaging and access to potentially curative options like resection. STRIDE’s OS advantage, despite PFS, supports this goal and contributed to its rapid inclusion as a category 1 recommendation in the NCCN guidelines following FDA approval [7].

Eligible patients in HIMALAYA had BCLC stage B or C disease, Child-Pugh class A, Eastern Cooperative Oncology Group (ECOG) performance status of 0 or 1, and measurable lesions per Response Evaluation Criteria in Solid Tumors (RECIST) v1.1, ensuring a representative cohort of advanced HCC [7]. The trial initially included four arms but was adjusted to three after finding no efficacy difference between durvalumab monotherapy and a lower-dose tremelimumab-durvalumab combination (T75 + D). STRIDE’s outcomes, compared to other regimens in Table 2, highlight its capacity to delay mortality while preserving liver function [7]. Nevertheless, the absence of PFS superiority raises important considerations regarding disease control and symptom burden between treatment intervals.

**Table 2:** Overall survival rates for STRIDE vs. sorafenib in the HIMALAYA trial [16]: highlighting STRIDE’s consistent survival advantage over time in unresectable HCC patients

Time point	STRIDE OS rate (95% CI)	Sorafenib OS rate (95% CI)	Absolute difference
**18 Months**	48.7% (43.6–53.5)	41.5% (36.5–46.4)	+7.2%
**24 Months**	40.5% (35.6–45.3)	32.6% (27.9–37.4)	+7.9%
**36 Months**	30.7% (25.8–35.7)	20.2% (15.8–25.1)	+10.5%

Note: OS, overall survival; CI, confidence interval; STRIDE, Single Tremelimumab Regular Interval Durvalumab; HIMALAYA, phase III randomized study that evaluated the STRIDE regimen; HCC, Hepatocellular carcinoma.

## 5 Safety and Adverse Events

The HIMALAYA trial assessed the safety profile of the STRIDE regimen using the National Cancer Institute Common Terminology Criteria for Adverse Events (version 4.03), and patient-reported outcomes via the European Organization for Research and Treatment of Cancer (EORTC) 30-item quality of life questionnaire. STRIDE significantly improved global health status scores, indicating a tolerable impact on patients’ overall quality of life despite its distinct toxicity profile [14].

Immune-mediated adverse events were notably more common in the STRIDE arm. High-dose steroids were required in 20.1% of patients (78/388), compared to 9.5% (37/389) with durvalumab monotherapy, 1.9% (7/393) with sorafenib, and 19.1% (29/152) with the T75 + D regimen. Treatment discontinuation due to immune-related adverse events occurred in 5.7% of STRIDE patients (22/388), vs. 2.6% (10/389) with durvalumab, 1.6% (6/393) with sorafenib, and 5.3% (8/152) with T75 + D [14].

Notably, STRIDE was associated with fewer hepatic and hemorrhagic events compared to sorafenib, a meaningful advantage in patients with underlying liver dysfunction [14]. However, the higher rate of immune-related toxicities, particularly those necessitating corticosteroids, requires regular monitoring of liver enzymes, thyroid function, and potential endocrinopathies every 4–6 weeks, following established ICI management guidelines [17]. These issues often demand multidisciplinary management, which may be particularly challenging in low-resource settings.

Furthermore, the development of treatment-induced anti-drug antibodies (anti-durvalumab and anti-tremelimumab) in some patients raises concerns about long-term immunogenicity and potential attenuation of efficacy. While these toxicities are generally manageable, they underscore the need for vigilant follow-up and appropriate supportive care. The incidence of immune-mediated toxicities, steroid requirements, and treatment discontinuation varied across treatment arms, with higher rates observed in STRIDE-based regimens (Table 3).

**Table 3:** Comparison of immune-mediated adverse events across HIMALAYA trial arms [14,18] highlights the balance between STRIDE’s efficacy and its tolerability profile

Treatment arm	Patients requiring high-dose steroids (%)	Discontinuation due to adverse events (%)
STRIDE [14]	20.1% (78/388)	5.7% (22/388)
Durvalumab [14]	9.5% (37/389)	2.6% (10/389)
Sorafenib [14]	1.9% (7/393)	1.6% (6/393)
T75 + D [14]	19.1% (29/152)	5.3% (8/152)

Note: STRIDE, Single Tremelimumab Regular Interval Durvalumab; T75 + D, durvalumab + tremelimumab.

## 6 Challenges in Treatment Selection

The past decade’s rapid evolution of systemic therapies for advanced HCC—from tyrosine kinase inhibitors like sorafenib to immunotherapy combinations like STRIDE and atezolizumab plus bevacizumab (A + B)—has broadened therapeutic options while complicating treatment selection. although these therapies aim to improve OS, PFS, and the potential for tumor downstaging, tailoring treatment to individual patients remains a significant challenge.

STRIDE’s OS advantage establishes it as a viable first-line choice, but its application requires careful consideration of patient-specific factors [7]. However, direct comparison with A + B, which demonstrated an updated OS of 19.2 months in the IMbrave150 trial, reveals important trade-offs. A + B carries a risk of VEGF-related bleeding, particularly in cirrhotic patients with esophageal or gastric varices [10]. Conversely, STRIDE presents a higher incidence of immune-mediated toxicities [14,18]. Nivolumab plus ipilimumab, recently approved as another dual immune checkpoint inhibitor (ICI) regimen, shows promising objective response rates (ORR) and durable responses, making it another potential option for patients with adequate hepatic function [19].

To mitigate bleeding risk with A + B, the IMbrave150 trial recommended esophagogastroduodenoscopy (EGD) within six months of treatment initiation. In that study, 41% of patients who underwent EGD had varices, and 19% required intervention [10]. However, real-world data from Canadian centers showed that 30% of A + B recipients omitted EGD—often those without cirrhosis or significant portal hypertension—without an observed increase in gastrointestinal bleeding (6% in the EGD group vs. 3% in the non-EGD group) [20]. These findings suggest that a selective EGD approach may be feasible, though standardized guidelines are lacking, especially for patients with prior variceal bleeding or progressive portal hypertension.

Despite the superior OS with A + B, STRIDE may be preferred in patients with contraindications to VEGF inhibition, recent variceal bleeding, or limited access to endoscopic surveillance. Its steroid-responsive immune toxicities may be easier to manage in multidisciplinary oncology settings compared to the vascular risks associated with bevacizumab [21,22].

Treatment selection should also incorporate emerging biomarkers such as PD-L1 expression, tumor mutational burden (TMB), and inflammatory gene signatures. These may help predict both ICI responsiveness and toxicity.

In non-viral HCC—such as those associated with non-alcoholic fatty liver disease (NAFLD)—preliminary evidence indicates reduced immunotherapy efficacy due to lower tumor immunogenicity. Proposed resistance mechanisms include T-cell exclusion, the presence of myeloid-derived suppressor cells (MDSCs), and the upregulation of alternative immune checkpoints. More robust data is needed to guide therapy in these populations [23,24].

For patients with impaired liver function, particularly Child-Pugh B7 or B8, early-phase trials and real-world studies suggest that monotherapy with ICIs may provide modest benefit with manageable toxicity. For example, in a prospective trial, nivolumab yielded a median OS of 7.6 months in Child-Pugh B patients. Similarly, retrospective analyses support the use of atezolizumab in select Child-Pugh B patients, albeit with reduced efficacy compared to those with Child-Pugh A [25]. Notably, the STRIDE regimen has only been studied in Child-Pugh A populations; therefore, its safety and efficacy in more advanced liver dysfunction remain unclear and require prospective investigation.

Radiation therapy also offers promising synergistic potential. External beam radiation can enhance the efficacy of TKIs and ICIs through mechanisms such as antigen presentation and activation of the STING pathway. Preclinical work by Dewan et al. demonstrated that radiation combined with CTLA-4 blockade led to superior tumor regression vs. either modality alone [26]. Furthermore, in a systematic review and meta-analysis. reported a median OS of 15.7 months in patients receiving external beam radiation plus sorafenib. These findings highlight radiation’s role as a potential adjunct in advanced HCC management [27].

Response heterogeneity remains a major challenge. Subgroup analyses indicate that non-viral HCC patients, such as those with NAFLD, may respond less effectively to STRIDE due to a less immunogenic tumor microenvironment. STRIDE’s immune-related toxicities also require comprehensive monitoring, which can be difficult in low-resource settings. Similarly, A + B’s bleeding risk, attributable to bevacizumab’s anti-VEGF action, necessitates close surveillance. Although some real-world data suggest that omitting routine EGD may be safe in low-risk patients, the absence of standardized guidelines introduces uncertainty in clinical decision-making.

Cost-effectiveness is another barrier. Both STRIDE and A + B are significantly more expensive than sorafenib, limiting their accessibility in resource-constrained settings, where HCC is often most prevalent [20]. Therefore, treatment selection must balance efficacy, toxicity, patient comorbidities, logistical feasibility, and economic considerations to achieve optimal outcomes.

Limitation:

This commentary is limited by its reliance on previously published studies, and no new data were generated for it.

## 7 Future Directions

The success of STRIDE highlights the promise of immunotherapy combinations in HCC, but challenges remain. The variability in patient responses, as seen with treatment-induced antibodies, suggests a need for biomarkers to identify ideal candidates and anticipate toxicity. Combining STRIDE with locoregional therapies such as TACE or radiation, particularly in borderline resectable cases, may improve outcomes through immune priming and tumor burden reduction, thereby improving downstaging outcomes. This integrative strategy could expand the pool of patients eligible for curative interventions such as resection or transplantation.

Ongoing and future clinical trials will likely refine STRIDE’s role, explore its use in broader populations (e.g., Child-Pugh B patients), and optimize combination regimens. Addressing immune-related adverse events through improved monitoring protocols and interdisciplinary management will be critical for wider adoption, especially in low-resource settings. As our understanding of tumor biology, immune modulation, and biomarker-driven therapy advances, treatment personalization will become central to improving outcomes in advanced HCC.

## 8 Conclusion

The STRIDE regimen represents a pivotal advancement in unresectable HCC management, offering a statistically significant OS improvement among approved systemic therapies. However, its modest survival benefit, coupled with higher rates of immune-related adverse events, necessitates nuanced patient selection and careful toxicity monitoring.

While its adoption as a standard of care reflects a favorable balance between efficacy and tolerability, STRIDE must be considered alongside other effective first-line options. It is not a one-size-fits-all solution. For clinicians, STRIDE offers a powerful tool to extend survival and potentially bridge selected patients to curative treatments, reinforcing its emerging role as a cornerstone of modern HCC therapy. Nevertheless, cost, toxicity, comorbidity burden, and evolving evidence must continue to guide personalized treatment planning.

## Data Availability

No new data was generated in this commentary. All information was discussed based on published studies, which are available in the reference.
